# Cytoplasmic Relocalization of TAR DNA-Binding Protein 43 Is Not Sufficient to Reproduce Cellular Pathologies Associated with ALS *In vitro*

**DOI:** 10.3389/fnmol.2017.00046

**Published:** 2017-02-24

**Authors:** Heike J. Wobst, Steven S. Wesolowski, Jayashree Chadchankar, Louise Delsing, Steven Jacobsen, Jayanta Mukherjee, Tarek Z. Deeb, John Dunlop, Nicholas J. Brandon, Stephen J. Moss

**Affiliations:** ^1^AstraZeneca-Tufts Laboratory for Basic and Translational Neuroscience, Department of Neuroscience, Tufts University School of Medicine, BostonMA, USA; ^2^IMED Biotech Unit, AstraZeneca Neuroscience IMED, AstraZeneca, CambridgeMA, USA; ^3^IMED Biotech Unit, AstraZeneca Discovery ScienceMölndal, Sweden; ^4^Department of Neuroscience, Tufts University School of Medicine, BostonMA, USA

**Keywords:** amyotrophic lateral sclerosis (ALS), TAR DNA-binding protein 43 (TDP-43), neurodegenerative diseases, frontotemporal dementia, mutation, protein misfolding disease

## Abstract

Mutations in the gene *TARDBP*, which encodes TAR DNA-binding protein 43 (TDP-43), are a rare cause of familial forms of amyotrophic lateral sclerosis (ALS) and frontotemporal dementia (FTD). While the majority of mutations are found in the C-terminal glycine-rich domain, an alanine to valine amino acid change at position 90 (A90V) in the bipartite nuclear localization signal (NLS) of TDP-43 has been described. This sequence variant has previously been shown to cause cytoplasmic mislocalization of TDP-43 and decrease protein solubility, leading to the formation of insoluble aggregates. Since the A90V mutation has been described both in patients as well as healthy controls, its pathogenic potential in ALS and FTD remains unclear. Here we compare properties of overexpressed A90V to the highly pathogenic M337V mutation. Though both mutations drive mislocalization of the protein to the cytoplasm to the same extent, M337V produces more significant damage in terms of protein solubility, levels of pathogenic phosphorylation, and formation of C-terminal truncated protein species. Furthermore, the M337V, but not the A90V mutant, leads to a downregulation of histone deacetylase 6 and Ras GTPase-activating protein-binding protein. We conclude that in the absence of another genetic or environmental ‘hit’ the A90V variant is not sufficient to cause the deleterious phenotypes associated with ALS and FTD, despite prominent cytoplasmic protein relocalization of TDP-43.

## Introduction

Amyotrophic lateral sclerosis (ALS) is a fast progressing neurodegenerative disorder associated with atrophy of upper and/or lower motor neurons leading to muscle wasting due to denervation. It is the third most frequently occurring neurodegenerative disease after Alzheimer’s disease and Parkinson’s disease (Hirtz et al., 2007). Death usually occurs within 2–3 years after diagnosis as a result of respiratory failure (Chio et al., 2011). Frontotemporal dementia (FTD) on the other hand is characterized by degeneration of neurons in the frontal and temporal lobes and is associated with impairment of cognitive abilities and language, as well as behavioral changes (Bang et al., 2015). Today, both diseases fall under the umbrella term of TDP-43 proteinopathies, in reference to the occurrence of cytoplasmic inclusions of the TAR DNA-binding protein 43 (TDP-43) in glial and neuronal cells: 97% of ALS as well as almost 50% of FTD cases present with such TDP-43-positive inclusions (Cairns et al., 2007; Mackenzie et al., 2007). In this pathological state, TDP-43 is found ubiquitinated, truncated, highly phosphorylated and aggregated (Arai et al., 2006; Neumann et al., 2006).

**FIGURE 1:**
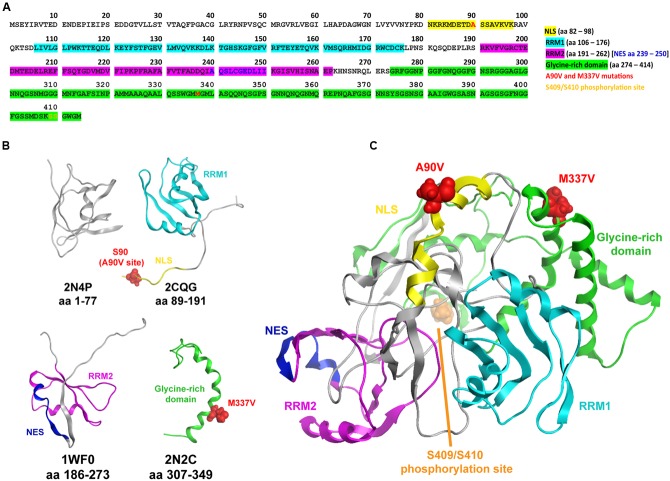
**Composite nuclear magnetic resonance (NMR) structure of TAR DNA-binding protein 43 (TDP-43). (A)** Human TDP-43 sequence illustrating the nuclear localization sequence (NLS), nuclear export signal (NES), RNA recognition motifs (RRM1 and RRM2), glycine-rich domain, as well as the A90V and M337V mutation sites, and the S409/S410 phosphorylation sites. **(B)** Available NMR solution structures for domains of TDP-43: PDB codes 2N4P, 2CQG, 1WF0, 2N2C (He et al., 2004; Suzuki et al., 2005; Lim and Song, 2015; Mompean et al., 2015; Lim et al., 2016; Mompean et al., 2016). Note that these structures – as well as the available X-ray structures 4IUF, 4Y00, and 4Y0F (Kuo et al., 2014; Chiang et al., 2016) – do not span the entire sequence, and large portions are either missing or poorly resolved, for example S90 in the largely unstructured terminus of 2CQG at the A90V site. **(C)** Composite TDP-43 schematic of available NMR structures. While iterative homology modeling and loop building techniques were used, a suitable template structure spanning across multiple domains was not found, and this composite structure should be considered as a schematic rather than a confident prediction of folding. Note that in this orientation the S409/S410 phosphorylation site is at the back of the structure near the N-terminus.

TAR DNA-binding protein 43 is a ubiquitously expressed RNA- and DNA-binding protein. In the central nervous system, TDP-43 binds over 6000 RNA targets. It has been shown to regulate the levels of 600 mRNA species and the alternative splicing of over 950 species (Polymenidou et al., 2011). RNA binding is mediated by two RNA recognition motifs, RRM1 and RRM2. In addition, the protein harbors a nuclear export signal (NES) as well as a bipartite NLS, and a C-terminal prion-like glycine-rich domain (**Figures 1A,C**) (Buratti and Baralle, 2001; Winton et al., 2008a). Mutations in the TDP-43 gene *TARDBP* in ALS were first described in 2008 (Gitcho et al., 2008; Kabashi et al., 2008; Sreedharan et al., 2008; Van Deerlin et al., 2008). The frequency of TDP-43 mutations is 1–5% in both sporadic and familial forms of ALS (Guerreiro et al., 2008; Kabashi et al., 2008; Kuhnlein et al., 2008; Iida et al., 2012; Zou et al., 2012).

The vast majority of *TARDBP* mutations are missense mutations, with all but three located in the C-terminal glycine-rich domain (Buratti, 2015). Two mutations, P112H and D169G, are located in the RRM1 domain (Kabashi et al., 2008; Buratti, 2015; Moreno et al., 2015). The third, an alanine to valine substitution at residue 90 (A90V), is found between the bipartite NLS (Winton et al., 2008b; Chiang et al., 2012). Winton et al. (2008b) showed that the A90V mutation leads to aberrant cytoplasmic localization and decreased solubility of TDP-43, two pathological hallmarks of TDP-43 proteinopathies, *in vitro*. Another study found that under stress conditions, induced pluripotent stem cells with the A90V mutation obtained from an FTD/ALS patient showed increased cell death, cytoplasmic localization of TDP-43 and downregulation of microRNA-9 (Zhang et al., 2013). However, the variant has been described both in FTD and ALS patients and control individuals (Guerreiro et al., 2008; Kabashi et al., 2008; Sreedharan et al., 2008; Winton et al., 2008b; Chiang et al., 2012). Further studies described the occurrence of the A90V variant at a higher frequency in Alzheimer’s disease patients compared to controls (Brouwers et al., 2010; Vanden Broeck et al., 2015). Thus the detection of the A90V variant in healthy controls combined with the biochemical findings reported by Winton et al. (2008b) leave its pathogenic nature unclear.

In this study, we compared the effects of the A90V variant of TDP-43 to both wild-type (WT) and the M337V mutation, a pathogenic mutation associated with familial ALS (Sreedharan et al., 2008), in the SH-SY5Y neuroblastoma cell line. We provide evidence that while the A90V amino acid change phenocopies the M337V mutation in terms of cytoplasmic mislocalization, it does not demonstrate such a severe phenotype in terms of formation of insoluble high molecular weight aggregates and C-terminal fragments, pathological phosphorylation or downregulation of TDP-43 targets G3BP and histone deacetylase 6 (HDAC6). We thus conclude that relocalization of TDP-43 from the nucleus to the cytoplasm alone, while a classic pathological feature of TDP-43 proteinopathies, is not in itself sufficient to cause the complete cellular disease signature. Furthermore, we propose that the A90V variant of TDP-43 could be a rare risk factor for ALS that requires another “hit” for the development of the full-blown disease pathology.

## Materials and Methods

### Plasmids

All plasmids were purchased from and customized by Origene. cDNA sequences were based on the accession number NM_007375.3 for human *TARDBP*. The WT construct was cloned into the pCMV6-AN-myc vector for expression of N-terminally tagged WT or mutant TDP-43 protein. Custom mutagenesis to generate the A90V (point mutation c.269C>T) and M337V (point mutation c.1009A>G) TDP-43 amino acid substitutions were carried out by Origene. The presence of each mutation was confirmed by DNA sequencing performed by GENEWIZ (forward sequencing primer 5′-GGACTTTCCAAAATGTCG-3′; reverse sequencing primer 5′-ATTAGGACAAGGCTGGTGGG-3′).

### Cell Culture

SH-SY5Y cells were grown at 37°C with 5% CO_2_ in a humidified atmosphere in minimum essential medium (MEM; Thermo Fisher Scientific) supplemented with L-glutamine, 10% fetal bovine serum and 1% penicillin/streptomycin. Cells were subcultured every 3–4 days. For immunofluorescence analysis, cells were grown on glass coverslips coated with 1 mg/mL poly-*L*-lysine (Sigma) in 24-well plates (Cellstar). For Western blot analysis, cells were grown in 6-well plates (Costar). Cells were transiently transfected using Fugene HD (Promega) following the manufacturer’s instructions 24 h after seeding (Fugene HD:DNA ratio 3:1). Total transfection time was 48 h.

### Antibodies

The following antibodies were used for immunofluorescence staining: rabbit N-terminal TDP-43 (1:500; 10782-2-AP, Proteintech), mouse anti-myc 9E10 (1:200; Developmental Studies Hybridoma Bank, 1:200: sc-40, Santa Cruz), mouse anti-scaffold attachment factor B (SAFB) 6F7 (1:300; ab8060, Abcam), mouse anti-protein-associated splicing factor (PSF) (1:50; P2860, Sigma), mouse anti-ubiquitin P4D1 (1:50; sc-8017, Santa Cruz). Anti-mouse and anti-rabbit secondary antibodies coupled to Alexa-488, Alexa-555, and Alexa-568 were used for detection (1:300; Thermo Fisher Scientific). For Western blot analysis, the following antibodies were used: rabbit C- and N-terminal TDP-43 (1:2,500; 12892-1-AP, 1:5,000; 10782-2-AP, Proteintech), mouse S409/410 phospho-TDP-43 (1:2,000; CAC-TIP-PTD-M01, Cosmo), mouse α-tubulin (1:15,000; ab80779, Abcam), rabbit HDAC6 (1:1,000; 12834-1-AP, Proteintech), mouse G3BP (1:1,000; 611126, BD Biosciences), mouse acetylated α-tubulin (1:3,000; 12152, Cell Signaling Technologies), mouse GAPDH 6C5 (1:2,000: sc-32233, Santa Cruz). Anti-mouse and anti-rabbit horseradish peroxidase-coupled secondary antibodies were purchased from Jackson Immunoresearch (1:7,000).

### Immunofluorescence Staining

Cells were washed in phosphate-buffered saline (PBS) and fixed in 4% paraformaldehyde (in PBS) for 15 min followed by permeabilization in 0.25% Triton-X (in PBS) for 10 min. Ten percent normal goat serum (in PBS; Abcam) was used as a blocking reagent. Cells were blocked for 1 h at room temperature and incubated overnight at 4°C in primary antibody solution. The next day, cells were washed with PBS and incubated for 1 h in secondary antibody solution. Coverslips were mounted with Prolong Gold Antifade Mountant with DAPI (Thermo Fisher Scientific). Image acquisition was performed using a Nikon A1 confocal/Eclipse Ti inverted microscope system and NIS Elements software (Nikon).

### Sequential Extraction of Insoluble Protein Aggregates

The extraction protocol was adapted from Cohen et al. (2012). Cells were grown in 6-well plates and transfected 24 h after seeding. After 48 h, transfected cells were lysed in 300 μL radio-immunoprecipitation assay (RIPA) buffer supplemented with 2 mM EDTA, 1 mM EGTA, protease inhibitors (Complete, Roche), and phosphatase inhibitors (PhosStop, Roche). Lysates were sonicated 2x 15 s with 20% maximum amplitude and centrifuged for 30 min at 100,000 × *g* and 4°C (Beckman Optima TLX ultracentrifuge with TLA100.3 rotor and Delrin adaptors). The supernatant was collected as the RIPA-soluble fraction. The pellet was washed in RIPA buffer and centrifuged for an additional 30 min at 100,000 × *g* and 4°C. The supernatant was discarded and the pellet was re-extracted in 100 μL urea buffer [7 M urea, 2 M thiourea, 30 mM Tris pH 8.5, 4% 3-[(3-Cholamidopropyl) dimethylammonio]-1-propanesulfonate (CHAPS, Sigma)]. The samples were sonicated 2x 15 s and centrifuged at room temperature for 30 min at 100,000 × *g*. The supernatant was collected as the urea-soluble fraction.

### Immunoblotting

Cells were lysed in RIPA buffer supplemented with 2 mM EDTA, 1 mM EGTA, protease inhibitors (Complete, Roche) and phosphatase inhibitors (PhosStop, Roche), and the protein concentration was determined by bicinchoninic acid (BCA) assay. RIPA and urea fractions obtained from sequential extractions were diluted 1:1 with RIPA buffer. Proteins were separated using standard Tris-glycine SDS-PAGE gels (polyacrylamide concentrations 8% for HDAC6, all others 10%) and transferred onto nitrocellulose (BioRad) or polyvinylidene difluoride (PVDF; Millipore) membranes. All blocking and antibody incubation steps were performed either in 5% milk in Tris-buffered saline (TBS) supplemented with 0.1% Tween-20 (TBS-T) or in 3% bovine serum albumin (BSA) in TBS-T. Western blots were developed with enhanced chemiluminescent substrates (ECL). Digital images were acquired with a ChemiDoc MP imaging system (BioRad) and quantified with Image Lab 5 (BioRad). Where necessary, blots were stripped with stripping buffer for 15 min (Restore, Thermo Fisher Scientific) and reprobed with loading control antibodies.

### Composite NMR Structure

The composite schematic of the full structure of human TDP-43 was produced from the nuclear magnetic resonance (NMR) structures 2N4P (Mompean et al., 2015, 2016), 2CQG (Suzuki et al., 2005), 1WF0 (He et al., 2004), and 2N2C (Lim and Song, 2015; Lim et al., 2016) [obtained from the Research Collaboratory for Structural Bioinformatics Protein Data Bank (RCSB PDB; Berman et al., 2000)] via iterative homology modeling and loop building using the Molecular Operating Environment (MOE) software (Molecular Operating Environment [MOE], 2017).

### Statistical Analysis

All data were expressed as mean ± standard error of the mean (SEM). Statistical analyses were performed using GraphPad Prism 6 software (San Diego, CA, USA). Normality was established using the Shapiro–Wilk test. Mean differences between groups were analyzed by one-way ANOVA and Tukey’s multiple comparisons test. The threshold for statistical significance was *p* ≤ 0.05. Experiments were replicated a minimum of three times.

## Results

### *In silico* Analysis of TDP-43 Protein and Determining Whether It Can Predict the Impact of Mutations on Protein Structure

Nuclear magnetic resonance and X-ray structural analysis of individual domains of WT and mutant TDP-43 continue to reveal insights into the relationships between disease-related mutations and the biophysical stability of the protein. We reasoned that assembling a three-dimensional model of TDP-43 may help us predict the structural impact of the A90V variant and support our *in vitro* efforts in elucidating its effects. Thus, we attempted to collate all structural information obtained from NMR and X-ray crystallography available in the public domain and obtain a complete 3D model of TDP-43 (**Figure 1**). However, structures including all domains which might enable a comprehensive examination of the effects of specific mutations on overall structure and stability are challenging and not yet realized. The human TDP-43 sequence (**Figure 1A**) and NMR structures (**Figure 1B**) in **Figure 1** highlight the NLS, NES, RRM1, RRM2, glycine-rich domain, as well as the A90V and M337V mutation sites and the S409/S410 phosphorylation site (He et al., 2004; Suzuki et al., 2005; Kuo et al., 2014; Lim and Song, 2015; Mompean et al., 2015, 2016; Chiang et al., 2016; Lim et al., 2016). The piecewise solutions of NMR and X-ray structures provide valuable domain-level information, but key portions of the protein remain either unresolved or missing. Iterative homology modeling and loop building techniques can be used to infer composite structures from their individual domains. However, in the case of TDP-43 a suitable template structure spanning across multiple domains does not exist, prohibiting the ability to confidently assess the overall structure (only an approximate schematic of the overall structure is shown, **Figure 1C**) and thus the impact of the A90V and M337V amino acid substitutions. Without *a priori* assumptions of the impact of the A90V variant based on a complete model, we then proceeded to investigate the consequences of expressing the A90V variant on common pathological phenotypes associated with disease: cytoplasmic relocalization, and the formation of insoluble TDP-43 and TDP-43 protein fragments. We contrasted the A90V variant with the well-characterized mutant M337V, which causes autosomal-dominant familial ALS (Sreedharan et al., 2008).

### The TDP-43 A90V Variant Phenocopies the M337V Variant by Causing Cytoplasmic Relocalization of TDP-43

**FIGURE 2:**
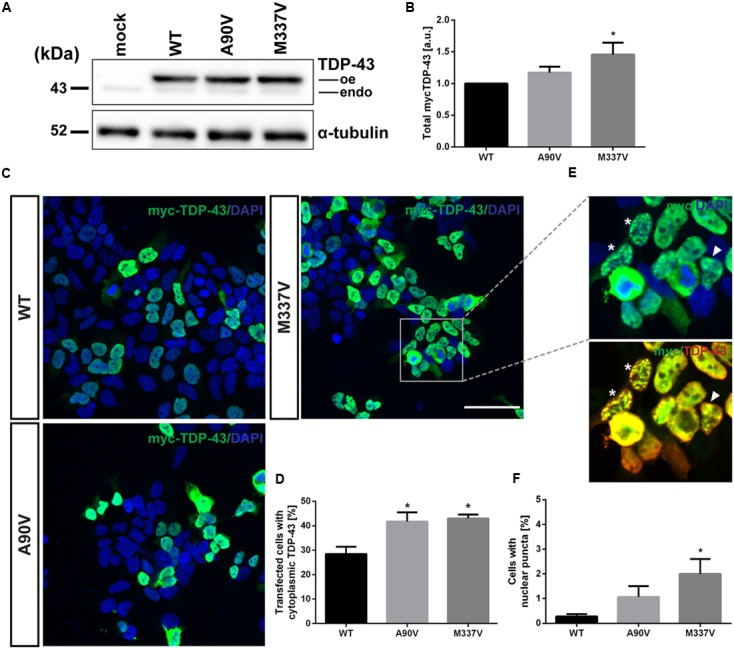
**The A90V and M337V variants of TDP-43 promote equivalent levels of cytoplasmic relocalization. (A)** Western blot of total overexpressed N-terminally myc-tagged TDP-43 WT, A90V and M337V (oe) and endogenous TDP-43 (endo). **(B)** Quantification of total TDP-43 levels (*N* = 6). **(C)** Immunofluorescence staining of SH-SY5Y cells transiently transfected with WT-, A90V- or M337V-TDP-43. Cells were co-stained with anti-myc (green) and anti-TDP-43 (red) antibodies. Nuclei were visualized with DAPI (blue). Scale bar = 30 μm. **(D)** Quantification of proportion of transfected cells with cytoplasmic TDP-43 localization. Results represent mean number of cells with cytoplasmic TDP-43 ± SEM (*N* = 3). **(E)** Immunofluorescence staining of TDP-43-positive nuclear puncta. Cells with multiple small inclusions or few larger inclusions are indicated with asterisk and arrowhead, respectively. **(F)** Quantification of transfected cells with punctate nuclear TDP-43 inclusions. Results represent mean number of transfected cells with inclusions ± SEM (*N* = 4).^∗^*p* < 0.05 (One-way ANOVA followed by Tukey’s multiple comparisons test).

According to Winton et al. (2008b), transient expression of the A90V variant of TDP-43 in QBI-293 cells results in the aberrant relocalization from the nucleus to the cytoplasm. We first verified expression of N-terminally myc-tagged TDP-43 constructs in SH-SY5Y cells by Western blot using an N-terminal TDP-43 antibody (**Figure 2A**). Steady state levels of total TDP-43 protein were significantly increased in cells expressing M337V mutant TDP-43 compared to WT expressing cells (0.46 ± 0.19-fold increase over WT; Tukey’s multiple comparisons analysis; *p* = 0.04), whereas there was no significant difference in cells expressing the A90V TDP-43 variant compared to WT protein (0.17 ± 0.09-fold over WT; Tukey’s multiple comparisons analysis; *p* = 0.57; **Figure 2B**; one-way ANOVA, *F*_2,15_ = 3.757; *P* = 0.0316). We confirmed that this increase in M337V TDP-43 protein levels was not caused by increased levels of mRNA, which were comparable between genotypes (Supplementary Figure S1).

We then performed immunofluorescence staining of transiently transfected cells in order to confirm the relocalization phenotype observed by Winton et al. (2008b) (**Figures 2C,D**). In agreement with the previous report (Winton et al., 2008b), we observed a significant increase in the proportion of cells with cytoplasmic TDP-43 localization in A90V, compared to WT TDP-43 expressing cells (WT: 28.4 ± 3.0% of cells; A90V: 41.7 ± 3.7% of cells; Tukey’s multiple comparisons analysis; *p* = 0.041). Interestingly, the proportion of transfected cells with cytoplasmic TDP-43 localization with A90V was very similar compared to the highly pathogenic mutant M337V (WT: 28.4 ± 3.0% of cells; M337V: 43.0 ± 1.6% of cells; Tukey’s multiple comparisons analysis; *p* = 0.028; one-way ANOVA *F*_2,6_ = 7.679; *P* = 0.0222; **Figure 2D**).

### The M337V Mutant, but Not the A90V Mutant, Enhances the Formation of Nuclear TDP-43 Inclusions

**FIGURE 3:**
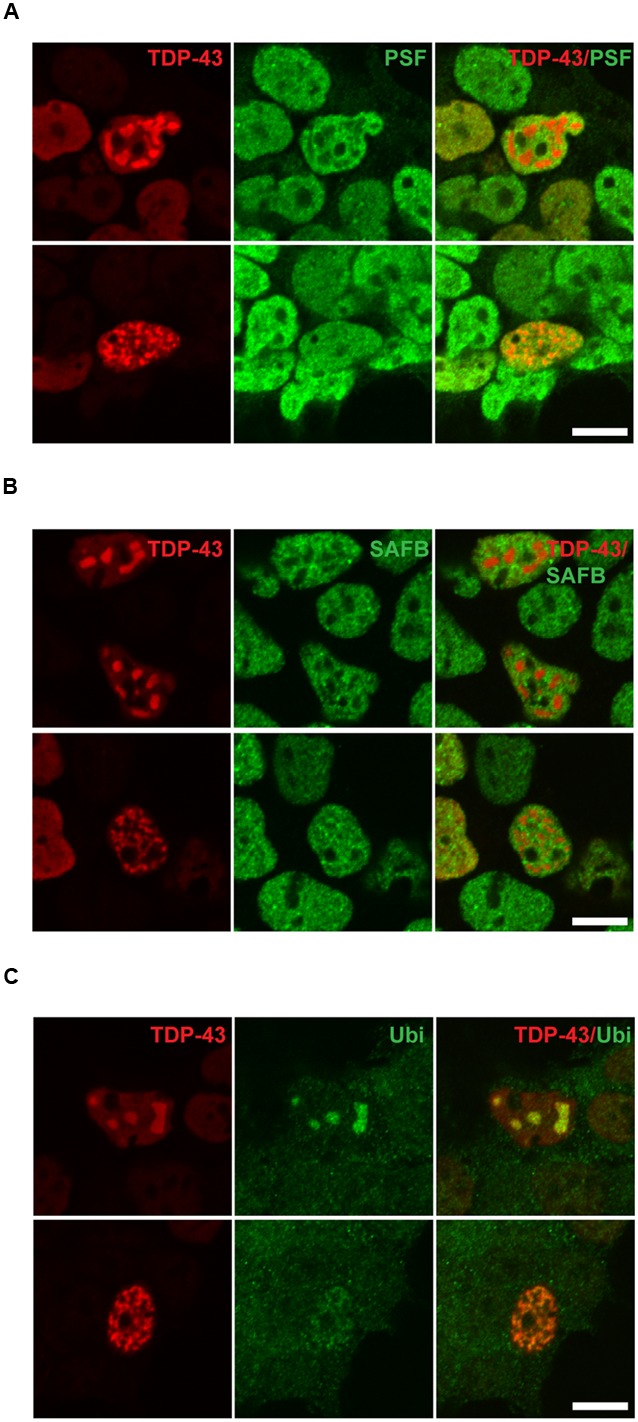
**TAR DNA-binding protein 43-positive nuclear puncta are inclusions of aggregated TDP-43, and do not co-localize with paraspeckles or nuclear stress bodies.** Co-localization of TDP-43-positive puncta with **(A)** paraspeckle marker polypyrimidine tract-binding protein-associated splicing factor (PSF), **(B)** nuclear stress body marker scaffold attachment factor B (SAFB), **(C)** ubiquitin. Left panels show representative images of cells with numerous small nuclear puncta, right panels cells with few, large puncta. All images shown represent M337V-transfected cells. Wild-type (WT) and A90V-transfected cells with puncta showed identical co-localization or lack thereof. Scale bar = 10 μm.

In a small number of transfected cells analyzed by immuno fluorescence staining we detected a punctate staining pattern for TDP-43 in the nucleus (**Figure 2E**). These TDP-43-positive puncta were only rarely observed in SH-SY5Y cells transfected with WT TDP-43 (0.28 ± 0.10% of transfected cells), but were found in significantly higher numbers in cells expressing the pathogenic mutant M337V (2.00 ± 0.60% of transfected cells; Tukey’s multiple comparisons analysis; *p* = 0.049; **Figure 2F**). However, we saw no significant difference in puncta formation between WT and A90V TDP-43 expressing cells (A90V: 1.06 ± 0.44% of transfected cells; Tukey’s multiple comparisons analysis; *p* = 0.44; one-way ANOVA *F*_2,9_ = 3.94; *P* = 0.059). In order to more closely investigate the composition of these TDP-43-containing puncta, we performed co-staining experiments with two nuclear markers: the nuclear stress body marker SAFB and the paraspeckle marker PSF (**Figure 3**). We qualitatively distinguished between transfected cells with small, numerous nuclear TDP-43-positive puncta (**Figure 2E**, asterisk; **Figures 3A–C**, lower panel) and cells with few, but large, puncta (**Figure 2E**, arrowhead; **Figures 3A–C**, upper panel). We observed no co-localization between TDP-43 protein and either SAFB or PSF in small puncta (**Figures 3A,B**). In cells harboring large TDP-43-positive puncta, both SAFB and PSF were found to be excluded from these structures. We hypothesized that these puncta represent intranuclear inclusions of insoluble, aggregated TDP-43 protein. As aggregated TDP-43 is found pathologically ubiquitinated (Neumann et al., 2006), we stained myc-TDP-43-transfected cells with an anti-ubiquitin antibody (**Figure 3C**). As expected, we detected diffuse ubiquitin staining in both the nucleus and cytoplasm. Both small and large TDP-43 nuclear puncta were shown to co-localize with ubiquitin, indicating that they are *bona fide* aggregates of ubiquitinated TDP-43 protein.

### The A90V TDP-43 Variant Has Subtle Effects on Protein Solubility and Pathologic Phosphorylation Compared to the M337V Mutation

**FIGURE 4:**
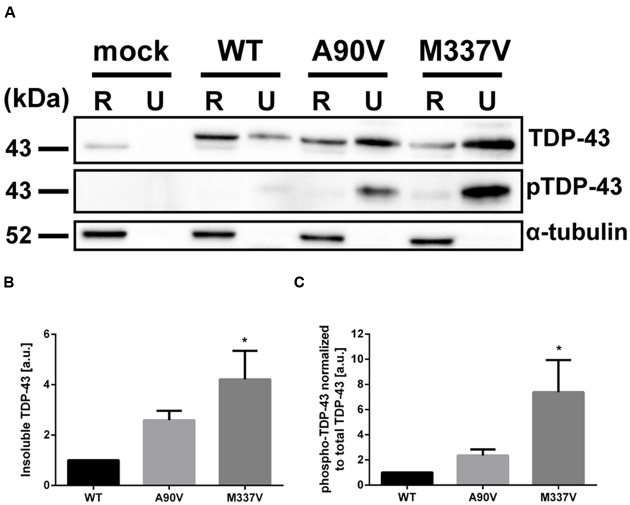
**The A90V TDP-43 variant has subtle effects on protein solubility and phosphorylation compared to the M337V variant. (A)** Western blot of total TDP-43 (N-terminal antibody) and phospho-S409/S410 TDP-43 in radio-immunoprecipitation assay (RIPA)-soluble (R) and RIPA-insoluble, urea-soluble (U) fractions. **(B,C)**, Western blot quantification of **(B)** total TDP-43 and **(C)** phospho-TDP-43 normalized to total TDP-43 in insoluble fractions. All results represent mean band signal ± SEM. ^∗^*p* < 0.05 (*N* = 4, One-way ANOVA followed by Tukey’s multiple comparisons test).

In order to further investigate how the A90V and M337V mutations affect the cell biology of TDP-43, we measured a range of cellular phenotypes which have been implicated in disease pathogenesis. Initially, we looked at the formation of insoluble TDP-43 aggregates. We fractionated lysates of transfected SH-SY5Y cells into RIPA-soluble and RIPA-insoluble, urea-soluble fractions by high speed centrifugation and separated proteins by SDS-PAGE. We used a TDP-43 antibody recognizing the N-terminus of TDP-43 to assess total TDP-43 protein levels in the RIPA and urea fractions (**Figure 4A**). In line with previous reports (Johnson et al., 2009; Bilican et al., 2012; Janssens et al., 2013), we found that the M337V mutation caused accumulation of insoluble TDP-43 in the urea fraction compared to WT (3.21 ± 1.14-fold increase over WT; Tukey’s multiple comparisons analysis; *p* = 0.023; one-way ANOVA *F*_2,9_ = 5.38; *P* = 0.029). While an enrichment of TDP-43 in the urea fraction was also observed in A90V-expressing cells (1.59 ± 0.38-fold increase compared to WT), the increase was not statistically significant (Tukey’s multiple comparisons analysis; *p* = 0.283; **Figure 4B**).

We used a phospho-specific TDP-43 antibody raised against the disease-relevant phosphorylation sites at S409/S410 (Neumann et al., 2009) to assess the phosphorylation status of the insoluble TDP-43 protein species (**Figure 4A**). We normalized the phospho-signal in the urea-soluble fractions to the total TDP-43 detected in these fractions to control for the relative increase in insoluble TDP-43 protein in mutant expressing cells (**Figure 4C**). Immunoblotting with this antibody revealed significantly increased phosphorylation levels at the S409/S410 residues in the urea-soluble fraction of M337V TDP-43 expressing cells compared to cells expressing WT TDP-43 (6.38 ± 2.56-fold increase compared to WT; Tukey’s multiple comparisons analysis; *p* = 0.036). We detected a non-statistically significant increase in S409/S410 phosphorylation in cells transfected with the A90V variant (1.35 ± 0.48-fold increase compared to WT; Tukey’s multiple comparisons analysis; *p* = 0.80; one-way ANOVA *F*_2,9_ = 5.015; *P* = 0.0344). Taken together, these results indicate that while the A90V and M337V mutations induce similar levels of cytoplasmic relocalization of TDP-43, a pathology commonly associated with disease, the effects of the M337V variant on protein solubility and phosphorylation are far greater than those of the A90V mutation. In fact there looks to be a spectrum of pathology in these transfected cell models with WT and M337V TDP-43 at the opposite extremes, with the A90V TDP-43 somewhere in between, in the absence of any additional stressors.

### M337V Mutant TDP-43 Readily Forms C-Terminal Fragments Compared to A90V and WT

**FIGURE 5:**
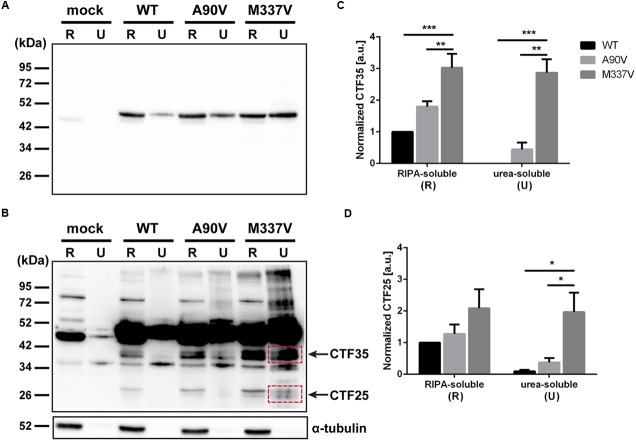
**The M337V TDP-43 variant readily forms C-terminal fragments compared to A90V and WT. (A,B)** Western blot of TDP-43 with C-terminal antibody for detection of C-terminal fragment. **(A)** short exposure, **(B)** longer exposure revealing 35 kDa fragment (CTF35) and 25 kDa fragment (CTF25). **(C,D)** Western blot quantification of TDP-43 fragment CTF35 **(C)** and CTF25 **(D)** (red boxes indicate quantified bands). All results represent mean band signal ± SEM. ^∗^*p* < 0.05, ^∗∗^*p* < 0.01, ^∗∗∗^*p* < 0.001 (*N* = 3, One-way ANOVA followed by Tukey’s multiple comparisons test).

In addition to abnormal phosphorylation as well as decreased protein solubility and aberrant cellular localization, another pathological feature of TDP-43 is the formation of C-terminal truncated protein fragments (Neumann et al., 2006; Mackenzie et al., 2007; Hasegawa et al., 2008). In order to detect C-terminal protein fragments not picked up by the N-terminal TDP-43 antibody used in **Figure 4** we extracted soluble and insoluble TDP-43 from transfected SH-SY5Y cells and used a C-terminal antibody (**Figures 5A,B**). We identified two smaller protein species present only in TDP-43-transfected cells, but not in the vector control cells (**Figure 5B**). The larger fragment ran close to the 34 kDa marker, while the smaller fragment ran close to the 26 kDa marker. C-terminal TDP-43 fragments with molecular weights of 35 kDa (CTF35) and 25 kDa (CTF25) have been described in a variety of cellular and animal models (Winton et al., 2008a; Xu et al., 2011; Bilican et al., 2012; Choksi et al., 2014; D’Alton et al., 2014; Li et al., 2015), and we will thus refer to the identified truncated TDP-43 species as CTF35 and CTF25. We quantified the amount of both fragments in the RIPA-soluble and urea-soluble fractions. We normalized to the amount of full-length TDP-43 in each sample in order to account for the increase of total TDP-43 protein. In the RIPA-soluble fraction, we observed an accumulation of CTF35 in M337V-transfected cells compared to either WT or A90V-TDP-43 (2.03 ± 0.44-fold increase over WT, *p* = 0.005; 0.68 ± 0.25-fold increase over A90V, *p* = 0.044; Tukey’s multiple comparisons analysis; one-way ANOVA *F*_2,6_ = 13.94; *P* = 0.0056; **Figure 5C**). However, we did not detect a significant increase of the smaller CTF25 in mutant-transfected cells (M337V: 1.09 ± 0.6-fold increase over WT, *p* = 0.19; 0.64 ± 0.52-fold increase over A90V, *p* = 0.36; Tukey’s multiple comparisons analysis; one-way ANOVA *F*_2,6_ = 2.166; *P* = 0.196). In the urea-soluble fractions the effect of the M337V mutant on the formation of these C-terminal fragments was even more striking: we detected a 20.0 ± 6.6-fold increase in the 25 kDa fragment in the M337V mutant compared to WT (Tukey’s multiple comparisons analysis; *p* = 0.026) and a 4.3 ± 1.7-fold increase compared to the A90V variant (Tukey’s multiple comparisons analysis; *p* = 0.049; one-way ANOVA *F*_2,6_ = 7.657; *P* = 0.0223; **Figure 5D**). We could not detect any 35 kDa fragment in the urea fraction of WT-TDP-43 transfected cells and found a 5.4 ± 1.1-fold increase in CTF35 levels in M337V- compared to A90V-transfected cells (M337V compared to WT: *p* = 0.0008: compared to A90V: *p* = 0.0019; Tukey’s multiple comparisons analysis; one-way ANOVA *F*_2,6_ = 31.53; *P* = 0.0007).

### The M337V TDP-43 Variant Down-regulates the Expression of G3BP and HDAC6

**FIGURE 6:**
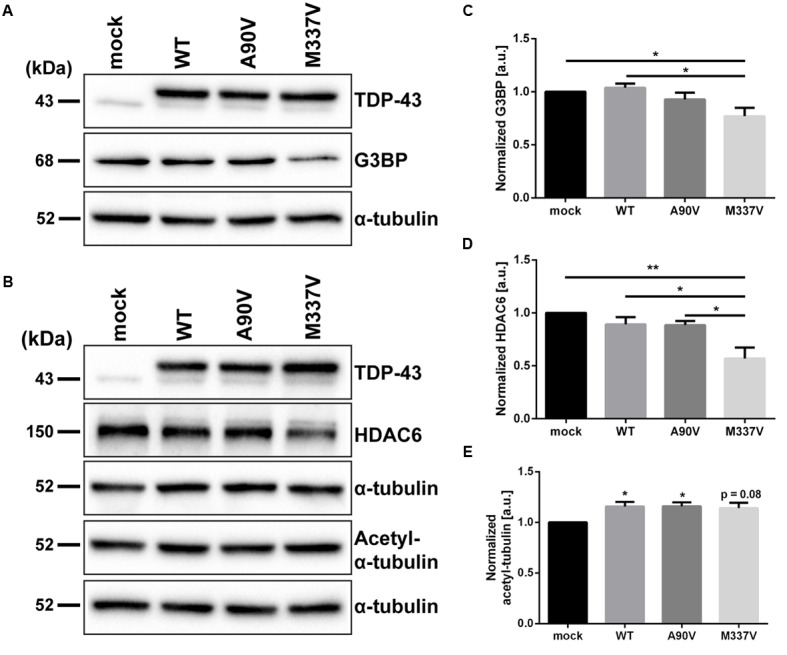
**The M337V, but not the A90V variant, leads to a downregulation of G3BP and HDAC6.** Western blots of **(A)** G3BP, **(B)** HDAC6 and acetylated α-tubulin levels in SH-SY5Y cells expressing WT, A90V and M337V TDP-43 or cells transfected with myc control vector. **(C–E)** Densitometry analyses of **(C)** G3BP (*N* = 6), **(D)** HDAC6 (*N* = 4), and **(E)** acetylated α-tubulin levels (*N* = 6). All results represent mean band signal ± SEM. ^∗^*p* < 0.05, ^∗∗^*p* < 0.01 (One-way ANOVA followed by Tukey’s multiple comparisons test).

We hypothesized that the M337V mutant of TDP-43 not only impacts TDP-43 localization and post-translational modifications in a manner more detrimental than the A90V variant, but that the downstream functional consequences of expressing M337V TDP-43 might reflect this more severe phenotype. Such a functional consequence could be altered regulation of transcriptional targets of TDP-43. Previous studies have shown that both depletion of endogenous TDP-43 as well as overexpression of R361S mutant TDP-43 lead to a decrease in G3BP, a regulator of stress granule assembly (McDonald et al., 2011; Aulas et al., 2012). We investigated expression levels of G3BP following 48 h of TDP-43 overexpression and found that only M337V, but not A90V, mutant TDP-43 significantly downregulated G3BP compared to WT TDP-43 and mock control (25.8 ± 8.3% compared to WT, *p* = 0.01; 23.0 ± 7.8% compared to mock, *p* = 0.03; Tukey’s multiple comparisons analysis; one-way ANOVA *F*_3,20_ = 4.861; *P* = 0.0106; **Figures 6A,C**). Furthermore, levels of HDAC6, a known target of TDP-43 that is likewise downregulated upon TDP-43 silencing (Fiesel et al., 2010), were significantly reduced in M337V TDP-43 expressing cells compared to cells transfected with WT and A90V TDP-43 or mock-transfected controls (WT: 36.1 ± 13.6%; *p* = 0.017; A90V: 35.6 ± 12.3%; *p* = 0.02; mock: 43.0 ± 10.2%; *p* = 0.002; Tukey’s multiple comparisons analysis; one-way ANOVA *F*_3,12_ = 8.455; *P* = 0.0027; **Figures 6B,D**). We also probed for levels of acetylated α-tubulin, a substrate for HDAC6 (Hubbert et al., 2002), but found no significant increase in acetylated α-tubulin levels in M337V-transfected cells compared to WT (*p* = 0.99) or A90V (*p* = 0.99) TDP-43 (Tukey’s multiple comparisons analysis; **Figures 6B,E**), suggesting that the decrease in HDAC6 protein levels in M337V-expressing cells is not sufficient to impair acetylation of α-tubulin. In comparison to mock-transfected controls, both WT and A90V-transfected cells showed a small, but significant upregulation of acetylated α-tubulin (WT: 15.8 ± 4.5%, *p* = 0.05; A90V: 16.0 ± 3.8%, *p* = 0.04; Tukey’s multiple comparisons analysis) and M337V-transfected cells showed a similar trend (14.2 ± 5.2%, *p* = 0.08; one-way ANOVA *F*_3,20_ = 3.833; *P* = 0.0256). An increase in α-tubulin acetylation has been described in conditions of cellular stress (Mackeh et al., 2014), which could be caused by the overexpression of TDP-43 irrespective of mutation. In conclusion, the M337V variant of TDP-43, but not the A90V variant, impacted the expression of known targets of TDP-43 in a manner consistent with the effects of a knockdown of TDP-43 function.

## Discussion

To date, genetic studies have revealed 52 missense mutations in the TDP-43 gene *TARDBP* as well as one insertion/deletion and one nonsense mutation (Buratti, 2015). While most mutations cluster in the C-terminal glycine-rich domain, the A90V amino acid exchange lies in the bipartite NLS. The pathogenic nature of this variant remains uncertain; *in vitro* studies suggest that A90V-TDP-43 is more prone to cytoplasmic mislocalization and aggregation, two pathological changes associated with ALS and FTD (Winton et al., 2008b). Furthermore, expression of A90V TDP-43 fails to fully ameliorate the pupal lethality and neuronal loss phenotypes caused by loss of the Drosophila ortholog of *TARDBP* (Vanden Broeck et al., 2015). Genetic studies, however, have revealed the presence of the A90V variant both in patients suffering from ALS and FTD as well as in healthy controls (Guerreiro et al., 2008; Kabashi et al., 2008; Sreedharan et al., 2008; Winton et al., 2008b; Chiang et al., 2012). It is thus likely that A90V is not a highly penetrant disease-associated mutation, but a genetic risk factor for TDP-43 proteinopathies. In order to gain a deeper understanding of the pathogenic potential of the A90V variant, we initially conducted analyses of available structural information for TDP-43 to see if we could generate hypotheses to test based on the site of the mutations. But unfortunately, due to the lack of complete NMR and X-ray structures, we were unable to predict the impact of the A90V variant of TDP-43 *in silico* (**Figure 1**). We then resorted to more traditional *in vitro* studies to measure TDP-43 mislocalization, changes in protein solubility and phosphorylation, and the effects on their expression on known targets of TDP-43. We compared the impact of A90V with that of the M337V mutation, a disease-associated mutation frequently used for *in vitro* and *in vivo* studies of ALS pathogenesis (Liachko et al., 2010; Zhou et al., 2010; Bilican et al., 2012; Janssens et al., 2013; Mutihac et al., 2015).

When we initially looked to reproduce the nuclear localization phenotype observed by Winton et al. (2008b) we found an almost identical increase in the number of cells with cytoplasmic TDP-43 with both the A90V and M337V variants. Thus, we concluded that the A90V variant of TDP-43 phenocopies the cytoplasmic relocalization phenotype associated with ALS found in the disease-associated M337V mutant. This strong effect is perhaps not surprising given the fact that the A90V variant lies within the bipartite NLS and could thus potentially interfere with nucleo-cytoplasmic shuttling of TDP-43.

In a small subset of transfected cells, we detected dot-like structures in the nucleus that stained positive for TDP-43. These TDP-43-positive nuclear structures were observed more frequently in cells transfected with the M337V, but not the A90V, mutant compared to WT. Instances of defined, patterned nuclear staining of TDP-43 *in vitro* have been described in the literature: overexpression of certain deletion mutants or the M337V mutant has been shown to cause the formation of nuclear inclusions of aggregated TDP-43 (Winton et al., 2008a; Nonaka et al., 2009a; Tripathi et al., 2014), while other papers describe co-localization of TDP-43 with nuclear bodies under certain circumstances, such as stress bodies under heat shock conditions, or NEAT1_2-containing paraspeckles (Nishimoto et al., 2013; Udan-Johns et al., 2014). We sought to define the nature of the observed nuclear TDP-43-positive puncta by performing co-localization experiments using antibodies against SAFB as a marker of nuclear stress bodies and PSF as a paraspeckle marker. We did not observe co-localization of either SAFB or PSF with TDP-43-positive inclusions, indicating that WT and mutant TDP-43 are not recruited into nuclear stress bodies or paraspeckles. However, we found that nuclear puncta of TDP-43 co-stain with ubiquitin, suggesting that they represent inclusions of aggregated, ubiquitinated TDP-43 in the nucleus. In this light, the increased frequency of these TDP-43-positive inclusions in the M337V mutant is probably the result of a higher aggregation propensity.

We investigated the aggregation propensity of the A90V and M337V mutants further by sequential extraction of total cell lysates. We found a stark increase in M337V in the insoluble fraction, in line with published literature showing increased aggregate formation of M337V-TDP-43 *in vitro*, *in vivo*, and in culture systems (Johnson et al., 2009; Zhou et al., 2010; Bilican et al., 2012). Compared to WT protein, the increase we observed in insoluble A90V TDP-43 was not statistically significant. Phosphorylation levels of insoluble, aggregated TDP-43 species at the pathologically relevant S409/410 residues were also markedly increased in the M337V mutant, while the effect in the A90V mutant was modest and not statistically significant. Analysis of total unfractionated lysate also revealed a significant increase in total mutant M337V TDP-43 protein compared to WT, likely due to the longer half-life of M337V-TDP-43 previously reported (Ling et al., 2010; Watanabe et al., 2013). While this increase in total M337V mutant protein could possibly help drive some of the described phenotypes, we believe the modest effect size (less than 50% compared to WT, less than 20% compared to A90V) is not sufficient to cause some of the more striking phenotypes we have observed, such as the effects on G3BP and HDAC6 expression levels.

C-terminally cleaved TDP-43 fragments are another pathological hallmark of ALS and FTD, and expression of TDP-43 in cell culture models can give rise to cleavage products of 35 and 25 kDa in the absence or presence of various stressors (Neumann et al., 2006; Hasegawa et al., 2008; Chiang et al., 2012; Yang et al., 2014; Li et al., 2015). We found an enrichment of two fragments of approximately 35 and 25 kDa in the insoluble fraction of M337V-transfected cells compared to both WT and A90V. Almost no C-terminal fragments were detected in the insoluble fraction of A90V-transfected cells, suggesting that A90V-TDP-43 is less prone to protease cleavage compared to the familial ALS mutant M337V. This phenotype is especially striking as several papers have suggested a crucial role for C-terminal TDP-43 fragments in enhancing cellular toxicity and aggregation propensity, especially the 25 kDa fragment (Johnson et al., 2008; Nonaka et al., 2009b; Zhang et al., 2009; Kitamura et al., 2016).

In addition to the effects of the A90V variant on TDP-43 aggregation, post-translational modifications and truncation, we investigated changes in known targets of TDP-43. We found that protein levels of G3BP are reduced in SH-SY5Y cells expressing M337V, but not A90V TDP-43. G3BP is a component of cytoplasmic stress granules, transient structures made from proteins and untranslated mRNAs, which form after insults such as heat shock and oxidative stress (Nover et al., 1989; Tourriere et al., 2003). Similarly, we found that only the M337V, but not the A90V mutant, leads to a reduction of the histone deacetylase HDAC6 in SH-SY5Y cells; HDAC6 has previously been claimed to be a critical component of stress granule assembly (Kwon et al., 2007). TDP-43 has been shown to be both recruited to and to regulate assembly of stress granules (Colombrita et al., 2009; McDonald et al., 2011). That the A90V variant of TDP-43, in contrast to the M337V variant, does not cause a downregulation of G3BP or HDAC6 could suggest that A90V does not interfere with stress granule assembly and dynamics, while the disease-associated M337V mutant does. In this regard, the M337V mutant causes a loss of normal function of TDP-43 regulation of stress granule assembly – while the A90V variant does not. Structurally, stress granules are protein-rich liquid droplets formed in a process termed liquid-liquid phase separation (LLPS). Recent studies have shown that mutations in the C-terminal domain, including the M337V mutation, inhibit this droplet formation by interrupting the interaction of α-helixes, driving the proteins to form aggregates instead (Molliex et al., 2015; Conicella et al., 2016; Schmidt and Rohatgi, 2016). Since the A90V variant is far removed from the α-helix in the C-terminal domain, it is likely that it does not interfere with the helical contact that drives droplet formation. However, further studies will be required to investigate whether A90V has more subtle effects on TDP-43 LLPS, droplet formation, and stress granule assembly.

In summary, the results of this study show that the A90V variant of TDP-43, which has been described as a rare variant found both in ALS and FTD patients and healthy controls, shows modest effects on protein solubility, phosphorylation, and truncation when compared to the disease-causing M337V mutation. In addition, in contrast to the M337V mutant, the A90V variant does not cause impairment of HDAC6 or G3BP expression. These findings are especially striking since we observed comparable levels of cytoplasmic TDP-43 relocalization in both A90V- and M337V-transfected cells, suggesting that cytoplasmic relocalization of TDP-43 protein is not by itself sufficient to cause the other molecular phenotypes associated with disease. Furthermore, we speculate that due to these modest *in vitro* effects observed with the A90V variant, carriers might not develop a disease phenotype in the absence of a “second hit.” However, it is possible that the A90V mutant could lower the threshold required for an additional stressor or other genetic risk factor to cause the catastrophic effects on TDP-43 and its functions observed in ALS and FTD. Thus, we conclude that the A90V variant of TDP-43 could be a rare genetic risk factor for TDP-43 proteinopathies. However, further studies will be required to gain an insight into the interplay between A90V as a genetic risk factor and other genetic and environmental risk factors associated with ALS and FTD.

## Author Contributions

HW and NB designed experiments; HW, JC, and LD carried out experiments; HW and JC analyzed the data; SW designed the composite NMR structure; JM and TD provided technical assistance; HW, NB, SW, and SM wrote the manuscript. All authors reviewed and approved the final version of the manuscript.

## Conflict of Interest Statement

SM serves as a consultant for AstraZeneca and SAGE Therapeutics in relationships that are regulated by Tufts University and do not impact on this study. At the time this work was conducted SW, SJ, JD, and NB were full-time employees and shareholders in AstraZeneca.

The other authors declare that the research was conducted in the absence of any commercial or financial relationships that could be construed as a potential conflict of interest.

## References

[B1] Arai T. Hasegawa M. Akiyama H. Ikeda K. Nonaka T. Mori H. (2006). TDP-43 is a component of ubiquitin-positive tau-negative inclusions in frontotemporal lobar degeneration and amyotrophic lateral sclerosis. *Biochem. Biophys. Res. Commun.* 351 602–611. 10.1016/j.bbrc.2006.10.093 17084815

[B2] Aulas A. Stabile S. Vande Velde C. (2012). Endogenous TDP-43, but not FUS, contributes to stress granule assembly via G3BP. *Mol. Neurodegener.* 7:54 10.1186/1750-1326-7-54 PMC3502460 23092511

[B3] Bang J. Spina S. Miller B. L. (2015). Frontotemporal dementia. *Lancet* 386 1672–1682. 10.1016/S0140-6736(15)00461-4 26595641 PMC5970949

[B4] Berman H. M. Westbrook J. Feng Z. Gilliland G. Bhat T. N. Weissig H. (2000). The protein data bank. *Nucleic Acids Res.* 28 235–242. 10592235 10.1093/nar/28.1.235 PMC102472

[B5] Bilican B. Serio A. Barmada S. J. Nishimura A. L. Sullivan G. J. Carrasco M. (2012). Mutant induced pluripotent stem cell lines recapitulate aspects of TDP-43 proteinopathies and reveal cell-specific vulnerability. *Proc. Natl. Acad. Sci. U.S.A.* 109 5803–5808. 10.1073/pnas.1202922109 22451909 PMC3326463

[B6] Brouwers N. Bettens K. Gijselinck I. Engelborghs S. Pickut B. A. Van Miegroet H. (2010). Contribution of TARDBP to Alzheimer’s disease genetic etiology. *J. Alzheimers Dis.* 21 423–430. 10.3233/JAD-2010-100198 20555136

[B7] Buratti E. (2015). Functional significance of TDP-43 mutations in disease. *Adv. Genet.* 91 1–53. 10.1016/bs.adgen.2015.07.001 26410029

[B8] Buratti E. Baralle F. E. (2001). Characterization and functional implications of the RNA binding properties of nuclear factor TDP-43, a novel splicing regulator of CFTR exon 9. *J. Biol. Chem.* 276 36337–36343. 10.1074/jbc.M104236200 11470789

[B9] Cairns N. J. Neumann M. Bigio E. H. Holm I. E. Troost D. Hatanpaa K. J. (2007). TDP-43 in familial and sporadic frontotemporal lobar degeneration with ubiquitin inclusions. *Am. J. Pathol.* 171 227–240. 10.2353/ajpath.2007.070182 17591968 PMC1941578

[B10] Chiang C. H. Grauffel C. Wu L. S. Kuo P. H. Doudeva L. G. Lim C. (2016). Structural analysis of disease-related TDP-43 D169G mutation: linking enhanced stability and caspase cleavage efficiency to protein accumulation. *Sci. Rep.* 6:21581 10.1038/srep21581 PMC4756693 26883171

[B11] Chiang H. H. Andersen P. M. Tysnes O. B. Gredal O. Christensen P. B. Graff C. (2012). Novel TARDBP mutations in Nordic ALS patients. *J. Hum. Genet.* 57 316–319. 10.1038/jhg.2012.24 22456481

[B12] Chio A. Calvo A. Moglia C. Mazzini L. Mora G. PARALS study group (2011). Phenotypic heterogeneity of amyotrophic lateral sclerosis: a population based study. *J. Neurol. Neurosurg. Psychiatry* 82 740–746. 10.1136/jnnp.2010.235952 21402743

[B13] Choksi D. K. Roy B. Chatterjee S. Yusuff T. Bakhoum M. F. Sengupta U. (2014). TDP-43 Phosphorylation by casein kinase Iepsilon promotes oligomerization and enhances toxicity in vivo. *Hum. Mol. Genet.* 23 1025–1035. 10.1093/hmg/ddt498 24105464

[B14] Cohen T. J. Hwang A. W. Unger T. Trojanowski J. Q. Lee V. M. (2012). Redox signalling directly regulates TDP-43 via cysteine oxidation and disulphide cross-linking. *EMBO J.* 31 1241–1252. 10.1038/emboj.2011.471 22193716 PMC3297986

[B15] Colombrita C. Zennaro E. Fallini C. Weber M. Sommacal A. Buratti E. (2009). TDP-43 is recruited to stress granules in conditions of oxidative insult. *J. Neurochem.* 111 1051–1061. 10.1111/j.1471-4159.2009.06383.x 19765185

[B16] Conicella A. E. Zerze G. H. Mittal J. Fawzi N. L. (2016). ALS mutations disrupt phase separation mediated by alpha-helical structure in the TDP-43 low-complexity C-terminal domain. *Structure* 24 1537–1549. 10.1016/j.str.2016.07.007 27545621 PMC5014597

[B17] D’Alton S. Altshuler M. Cannon A. Dickson D. W. Petrucelli L. Lewis J. (2014). Divergent phenotypes in mutant TDP-43 transgenic mice highlight potential confounds in TDP-43 transgenic modeling. *PLoS ONE* 9:e86513 10.1371/journal.pone.0086513 PMC3899264 24466128

[B18] Fiesel F. C. Voigt A. Weber S. S. Van den Haute C. Waldenmaier A. Gorner K. (2010). Knockdown of transactive response DNA-binding protein (TDP-43) downregulates histone deacetylase 6. *EMBO J.* 29 209–221. 10.1038/emboj.2009.324 19910924 PMC2808372

[B19] Gitcho M. A. Baloh R. H. Chakraverty S. Mayo K. Norton J. B. Levitch D. (2008). TDP-43 A315T mutation in familial motor neuron disease. *Ann. Neurol.* 63 535–538. 10.1002/ana.21344 18288693 PMC2747362

[B20] Guerreiro R. J. Schymick J. C. Crews C. Singleton A. Hardy J. Traynor B. J. (2008). TDP-43 is not a common cause of sporadic amyotrophic lateral sclerosis. *PLoS ONE* 3:e2450 10.1371/journal.pone.0002450 PMC2408729 18545701

[B21] Hasegawa M. Arai T. Nonaka T. Kametani F. Yoshida M. Hashizume Y. (2008). Phosphorylated TDP-43 in frontotemporal lobar degeneration and amyotrophic lateral sclerosis. *Ann. Neurol.* 64 60–70. 10.1002/ana.21425 18546284 PMC2674108

[B22] He F. Muto Y. Inoue M. Kigawa T. Shirouzu M. Terada T. (2004). PDB ID: 1WF0–Solution structure of RRM domain in TAR DNA-binding protein-43. 10.2210/pdb1wf0/pdb

[B23] Hirtz D. Thurman D. J. Gwinn-Hardy K. Mohamed M. Chaudhuri A. R. Zalutsky R. (2007). How common are the "common" neurologic disorders? *Neurology* 68 326–337. 10.1212/01.wnl.0000252807.38124.a3 17261678

[B24] Hubbert C. Guardiola A. Shao R. Kawaguchi Y. Ito A. Nixon A. (2002). HDAC6 is a microtubule-associated deacetylase. *Nature* 417 455–458. 10.1038/417455a 12024216

[B25] Iida A. Kamei T. Sano M. Oshima S. Tokuda T. Nakamura Y. (2012). Large-scale screening of TARDBP mutation in amyotrophic lateral sclerosis in Japanese. *Neurobiol. Aging* 33 786–790. 10.1016/j.neurobiolaging.2010.06.017 20675015

[B26] Janssens J. Wils H. Kleinberger G. Joris G. Cuijt I. Ceuterick-de Groote C. (2013). Overexpression of ALS-associated p.*M337V* human TDP-43 in mice worsens disease features compared to wild-type human TDP-43 mice. *Mol. Neurobiol.* 48 22–35. 10.1007/s12035-013-8427-5 23475610 PMC3718993

[B27] Johnson B. S. McCaffery J. M. Lindquist S. Gitler A. D. (2008). A yeast TDP-43 proteinopathy model: exploring the molecular determinants of TDP-43 aggregation and cellular toxicity. *Proc. Natl. Acad. Sci. U.S.A.* 105 6439–6444. 10.1073/pnas.0802082105 18434538 PMC2359814

[B28] Johnson B. S. Snead D. Lee J. J. McCaffery J. M. Shorter J. Gitler A. D. (2009). TDP-43 is intrinsically aggregation-prone, and amyotrophic lateral sclerosis-linked mutations accelerate aggregation and increase toxicity. *J. Biol. Chem.* 284 20329–20339. 10.1074/jbc.M109.010264 19465477 PMC2740458

[B29] Kabashi E. Valdmanis P. N. Dion P. Spiegelman D. McConkey B. J. Vande Velde C. (2008). TARDBP mutations in individuals with sporadic and familial amyotrophic lateral sclerosis. *Nat. Genet.* 40 572–574. 10.1038/ng.132 18372902

[B30] Kitamura A. Nakayama Y. Shibasaki A. Taki A. Yuno S. Takeda K. (2016). Interaction of RNA with a C-terminal fragment of the amyotrophic lateral sclerosis-associated TDP43 reduces cytotoxicity. *Sci. Rep.* 6:19230 10.1038/srep19230 PMC4725827 26757674

[B31] Kuhnlein P. Sperfeld A. D. Vanmassenhove B. Van Deerlin V. Lee V. M. Trojanowski J. Q. (2008). Two German kindreds with familial amyotrophic lateral sclerosis due to TARDBP mutations. *Arch. Neurol.* 65 1185–1189. 10.1001/archneur.65.9.1185 18779421 PMC2742976

[B32] Kuo P. H. Chiang C. H. Wang Y. T. Doudeva L. G. Yuan H. S. (2014). The crystal structure of TDP-43 RRM1-DNA complex reveals the specific recognition for UG- and TG-rich nucleic acids. *Nucleic Acids Res.* 42 4712–4722. 10.1093/nar/gkt1407 24464995 PMC3985631

[B33] Kwon S. Zhang Y. Matthias P. (2007). The deacetylase HDAC6 is a novel critical component of stress granules involved in the stress response. *Genes Dev.* 21 3381–3394. 10.1101/gad.461107 18079183 PMC2113037

[B34] Li Q. Yokoshi M. Okada H. Kawahara Y. (2015). The cleavage pattern of TDP-43 determines its rate of clearance and cytotoxicity. *Nat. Commun.* 6:6183 10.1038/ncomms7183 25630387

[B35] Liachko N. F. Guthrie C. R. Kraemer B. C. (2010). Phosphorylation promotes neurotoxicity in a *Caenorhabditis elegans* model of TDP-43 proteinopathy. *J. Neurosci.* 30 16208–16219. 10.1523/JNEUROSCI.2911-10.2010 21123567 PMC3075589

[B36] Lim L. Song J. (2015). PDB ID: 2N2C–ALS-causing mutations significantly perturb the self-assembly and interaction with nucleic acid of the intrinsically-disordered prion-like domain of TDP-43. 10.2210/pdb2n2c/pdb PMC4703307 26735904

[B37] Lim L. Wei Y. Lu Y. Song J. (2016). ALS-causing mutations significantly perturb the self-assembly and interaction with nucleic acid of the intrinsically disordered prion-like domain of TDP-43. *PLoS Biol.* 14:e1002338 10.1371/journal.pbio.1002338 PMC4703307 26735904

[B38] Ling S. C. Albuquerque C. P. Han J. S. Lagier-Tourenne C. Tokunaga S. Zhou H. (2010). ALS-associated mutations in TDP-43 increase its stability and promote TDP-43 complexes with FUS/TLS. *Proc. Natl. Acad. Sci. U.S.A.* 107 13318–13323. 10.1073/pnas.1008227107 20624952 PMC2922163

[B39] Mackeh R. Lorin S. Ratier A. Mejdoubi-Charef N. Baillet A. Bruneel A. (2014). Reactive oxygen species, AMP-activated protein kinase, and the transcription cofactor p300 regulate alpha-tubulin acetyltransferase-1 (alphaTAT-1/MEC-17)-dependent microtubule hyperacetylation during cell stress. *J. Biol. Chem.* 289 11816–11828. 10.1074/jbc.M113.507400 24619423 PMC4002089

[B40] Mackenzie I. R. Bigio E. H. Ince P. G. Geser F. Neumann M. Cairns N. J. (2007). Pathological TDP-43 distinguishes sporadic amyotrophic lateral sclerosis from amyotrophic lateral sclerosis with SOD1 mutations. *Ann. Neurol.* 61 427–434. 10.1002/ana.21147 17469116

[B41] McDonald K. K. Aulas A. Destroismaisons L. Pickles S. Beleac E. Camu W. (2011). TAR DNA-binding protein 43 (TDP-43) regulates stress granule dynamics via differential regulation of G3BP and TIA-1. *Hum. Mol. Genet.* 20 1400–1410. 10.1093/hmg/ddr021 21257637

[B42] Molecular Operating Environment [MOE] (2017). *Molecular Operating Environment, 2013.08*. Montreal, QC: Chemical Computing Group Inc.

[B43] Molliex A. Temirov J. Lee J. Coughlin M. Kanagaraj A. P. Kim H. J. (2015). Phase separation by low complexity domains promotes stress granule assembly and drives pathological fibrillization. *Cell* 163 123–133. 10.1016/j.cell.2015.09.015 26406374 PMC5149108

[B44] Mompean M. Romano V. Pantoja-Uceda D. Stuani C. Baralle F. Buratti E. (2015). PDB ID: 2N4P–solution structure of the N-Terminal domain of TDP-43. 10.2210/pdb2n4p/pdb

[B45] Mompean M. Romano V. Pantoja-Uceda D. Stuani C. Baralle F. E. Buratti E. (2016). The TDP-43 N-terminal domain structure at high resolution. *FEBS J.* 283 1242–1260. 10.1111/febs.13651 26756435

[B46] Moreno F. Rabinovici G. D. Karydas A. Miller Z. Hsu S. C. Legati A. (2015). A novel mutation P112H in the TARDBP gene associated with frontotemporal lobar degeneration without motor neuron disease and abundant neuritic amyloid plaques. *Acta Neuropathol. Commun.* 3 19 10.1186/s40478-015-0190-6 PMC4382926 25853458

[B47] Mutihac R. Alegre-Abarrategui J. Gordon D. Farrimond L. Yamasaki-Mann M. Talbot K. (2015). TARDBP pathogenic mutations increase cytoplasmic translocation of TDP-43 and cause reduction of endoplasmic reticulum Ca(2)(+) signaling in motor neurons. *Neurobiol. Dis.* 75 64–77. 10.1016/j.nbd.2014.12.010 25526708

[B48] Neumann M. Kwong L. K. Lee E. B. Kremmer E. Flatley A. Xu Y. (2009). Phosphorylation of S409/410 of TDP-43 is a consistent feature in all sporadic and familial forms of TDP-43 proteinopathies. *Acta Neuropathol.* 117 137–149. 10.1007/s00401-008-0477-9 19125255 PMC2693625

[B49] Neumann M. Sampathu D. M. Kwong L. K. Truax A. C. Micsenyi M. C. Chou T. T. (2006). Ubiquitinated TDP-43 in frontotemporal lobar degeneration and amyotrophic lateral sclerosis. *Science* 314 130–133. 10.1126/science.1134108 17023659

[B50] Nishimoto Y. Nakagawa S. Hirose T. Okano H. J. Takao M. Shibata S. (2013). The long non-coding RNA nuclear-enriched abundant transcript 1_2 induces paraspeckle formation in the motor neuron during the early phase of amyotrophic lateral sclerosis. *Mol. Brain* 6:31 10.1186/1756-6606-6-31 PMC3729541 23835137

[B51] Nonaka T. Arai T. Buratti E. Baralle F. E. Akiyama H. Hasegawa M. (2009a). Phosphorylated and ubiquitinated TDP-43 pathological inclusions in ALS and FTLD-U are recapitulated in SH-SY5Y cells. *FEBS Lett.* 583 394–400. 10.1016/j.febslet.2008.12.031 19111550

[B52] Nonaka T. Kametani F. Arai T. Akiyama H. Hasegawa M. (2009b). Truncation and pathogenic mutations facilitate the formation of intracellular aggregates of TDP-43. *Hum. Mol. Genet.* 18 3353–3364. 10.1093/hmg/ddp275 19515851

[B53] Nover L. Scharf K. D. Neumann D. (1989). Cytoplasmic heat shock granules are formed from precursor particles and are associated with a specific set of mRNAs. *Mol. Cell. Biol.* 9 1298–1308. 10.1128/MCB.9.31298 2725500 PMC362722

[B54] Polymenidou M. Lagier-Tourenne C. Hutt K. R. Huelga S. C. Moran J. Liang T. Y. (2011). Long pre-mRNA depletion and RNA missplicing contribute to neuronal vulnerability from loss of TDP-43. *Nat. Neurosci.* 14 459–468. 10.1038/nn.2779 21358643 PMC3094729

[B55] Schmidt H. B. Rohatgi R. (2016). In Vivo formation of vacuolated multi-phase compartments lacking membranes. *Cell Rep.* 16 1228–1236. 10.1016/j.celrep.2016.06.088 27452472 PMC4972689

[B56] Sreedharan J. Blair I. P. Tripathi V. B. Hu X. Vance C. Rogelj B. (2008). TDP-43 mutations in familial and sporadic amyotrophic lateral sclerosis. *Science* 319 1668–1672. 10.1126/science.1154584 18309045 PMC7116650

[B57] Suzuki S. Muto Y. Inoue M. Kigawa T. Terada T. Shirouzu M. (2005). PDB ID: 2CQG–solution structure of the RNA binding domain of TAR DNA-binding protein-43. 10.2210/pdb2cqg/pdb

[B58] Tourriere H. Chebli K. Zekri L. Courselaud B. Blanchard J. M. Bertrand E. (2003). The RasGAP-associated endoribonuclease G3BP assembles stress granules. *J. Cell Biol.* 160 823–831. 10.1083/jcb.200212128 12642610 PMC2173781

[B59] Tripathi V. B. Baskaran P. Shaw C. E. Guthrie S. (2014). Tar DNA-binding protein-43 (TDP-43) regulates axon growth in vitro and in vivo. *Neurobiol. Dis.* 65 25–34. 10.1016/j.nbd.2014.01.004 24423647 PMC3988849

[B60] Udan-Johns M. Bengoechea R. Bell S. Shao J. Diamond M. I. True H. L. (2014). Prion-like nuclear aggregation of TDP-43 during heat shock is regulated by HSP40/70 chaperones. *Hum. Mol. Genet.* 23 157–170. 10.1093/hmg/ddt408 23962724 PMC3857952

[B61] Van Deerlin V. M. Leverenz J. B. Bekris L. M. Bird T. D. Yuan W. Elman L. B. (2008). TARDBP mutations in amyotrophic lateral sclerosis with TDP-43 neuropathology: a genetic and histopathological analysis. *Lancet Neurol.* 7 409–416. 10.1016/S1474-4422(08)70071-1 18396105 PMC3546119

[B62] Vanden Broeck L. Kleinberger G. Chapuis J. Gistelinck M. Amouyel P. Van Broeckhoven C. (2015). Functional complementation in *Drosophila* to predict the pathogenicity of TARDBP variants: evidence for a loss-of-function mechanism. *Neurobiol. Aging* 36 1121–1129. 10.1016/j.neurobiolaging.2014.09.001 25442115

[B63] Watanabe S. Kaneko K. Yamanaka K. (2013). Accelerated disease onset with stabilized familial amyotrophic lateral sclerosis (ALS)-linked mutant TDP-43 proteins. *J. Biol. Chem.* 288 3641–3654. 10.1074/jbc.M112.433615 23235148 PMC3561582

[B64] Winton M. J. Igaz L. M. Wong M. M. Kwong L. K. Trojanowski J. Q. Lee V. M. (2008a). Disturbance of nuclear and cytoplasmic TAR DNA-binding protein (TDP-43) induces disease-like redistribution, sequestration, and aggregate formation. *J. Biol. Chem.* 283 13302–13309. 10.1074/jbc.M800342200 18305110 PMC2442318

[B65] Winton M. J. Van Deerlin V. M. Kwong L. K. Yuan W. Wood E. M. Yu C. E. (2008b). A90V TDP-43 variant results in the aberrant localization of TDP-43 in vitro. *FEBS Lett.* 582 2252–2256. 10.1016/j.febslet.2008.05.024 18505686 PMC2478749

[B66] Xu Y. F. Zhang Y. J. Lin W. L. Cao X. Stetler C. Dickson D. W. (2011). Expression of mutant TDP-43 induces neuronal dysfunction in transgenic mice. *Mol. Neurodegener.* 6:73 10.1186/1750-1326-6-73 PMC3216869 22029574

[B67] Yang Z. Lin F. Robertson C. S. Wang K. K. (2014). Dual vulnerability of TDP-43 to calpain and caspase-3 proteolysis after neurotoxic conditions and traumatic brain injury. *J. Cereb. Blood Flow Metab.* 34 1444–1452. 10.1038/jcbfm.2014.105 24917042 PMC4158661

[B68] Zhang Y. J. Xu Y. F. Cook C. Gendron T. F. Roettges P. Link C. D. (2009). Aberrant cleavage of TDP-43 enhances aggregation and cellular toxicity. *Proc. Natl. Acad. Sci. U.S.A.* 106 7607–7612. 10.1073/pnas.0900688106 19383787 PMC2671323

[B69] Zhang Z. Almeida S. Lu Y. Nishimura A. L. Peng L. Sun D. (2013). Downregulation of microRNA-9 in iPSC-derived neurons of FTD/ALS patients with TDP-43 mutations. *PLoS ONE* 8:e76055 10.1371/journal.pone.0076055 PMC3797144 24143176

[B70] Zhou H. Huang C. Chen H. Wang D. Landel C. P. Xia P. Y. (2010). Transgenic rat model of neurodegeneration caused by mutation in the TDP gene. *PLoS Genet.* 6:e1000887 10.1371/journal.pgen.1000887 PMC2845661 20361056

[B71] Zou Z. Y. Peng Y. Wang X. N. Liu M. S. Li X. G. Cui L. Y. (2012). Screening of the TARDBP gene in familial and sporadic amyotrophic lateral sclerosis patients of Chinese origin. *Neurobiol. Aging* 33 2229.e11–e2229.e18. 10.1016/j.neurobiolaging.2012.03.014 22575358

