# Supplementary material for: Cytoplasmic Relocalization of TAR DNA-Binding Protein 43 Is Not Sufficient to Reproduce Cellular Pathologies Associated with ALS In vitro
Source: Front Mol Neurosci. 2017 Feb 24;10:46. doi: 10.3389/fnmol.2017.00046 (PMC5323424; doi:10.3389/fnmol.2017.00046)

**Supplementary info**

**Methods**

***Quantitative PCR***

RNA was extracted from SH-SY5Y cells using the RNeasy Plus Mini Kit (Qiagen). CDNA was synthesized using SuperScript® III First-Strand Synthesis kit (Life Technologies) with random hexamers following manufacturer’s instructions. For qPCR, cDNA was amplified using 600nM primers and 2x SYBR Green master mix in a volume of 25 µl in the Mx3000P system (Agilent) in triplicates using the following primers:

*TDP-43*

Forward 5’ TATGGGTGGTGGGATGAACT Reverse 5’ GCCTCCATTAAAACCACTGC

*TUBB*

Forward 5’ CAGAGCGGTGCTGGTGGAC Reverse 5’ GAGGGCACCACGCTGAAG

The gene dose was calculated based on the standard curve method relative to *TUBB* and normalized to mean control values.

**Results**

**Supplementary Figure 1 mRNA levels of TDP-43 in SH-SY5Y cells transiently transfected with control plasmid (mock), TDP-43 WT, TDP-43 A90V or TDP-43 M337V.** mRNA levels were normalized to TUBB. Results represent mean ± S.D. (N = 3). One-way ANOVA: F_3,8_ = 2.138, P = 0.1735


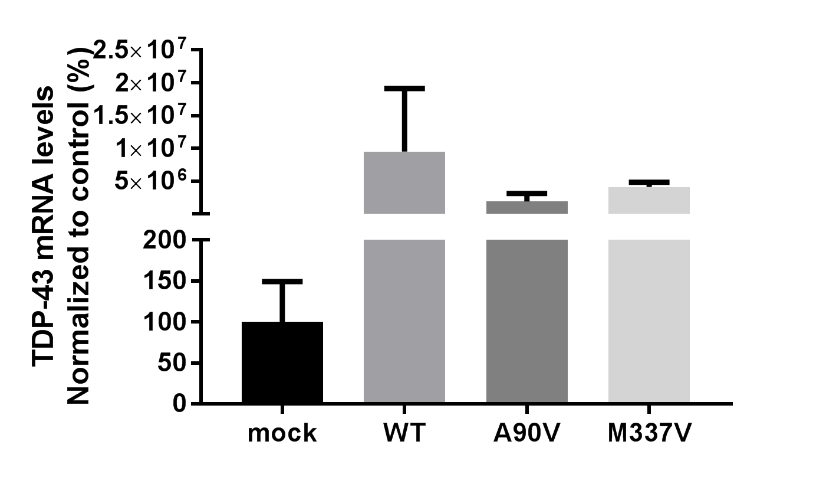

Supplement: Supplementary file 1 [file Data_Sheet_1.DOCX]
